# Editorial: The functional plasticity of innate immune cells

**DOI:** 10.3389/fimmu.2026.1993921

**Published:** 2026-09-29

**Authors:** Filipe Rodolfo Moreira Borges Oliveira, Jacqueline Alves Leite, Luiz Henrique Agra Cavalcante-Silva

**Affiliations:** 1 Department of Biomedical and Molecular Sciences, Queen’s University, Kingston, ON, Canada; 2 Institute of Biological Sciences, Department of Pharmacology, Federal University of Goiás, Goiânia, Brazil; 3 Medical Sciences and Nursing Complex, Federal University of Alagoas, Arapiraca, Brazil

**Keywords:** dendritic cell, HIV, iNKT cell, innate lymphoid cell, macrophage, neutrophil, NK cell, γδ T cells

From early observations of phagocytic activity by Metchnikov in the late 19th century to the identification of distinct innate lymphoid cell subsets in the early twenty-first century, successive discoveries have revealed the remarkable functional diversity of the innate immune system. Rather than being fixed or confined to specific cell types, these functions are shaped by tissue context, cellular metabolism, and microenvironmental cues. This functional plasticity establishes innate immune cells as key players and potential therapeutic targets in multiple immune-mediated diseases. In the Research Topic “*The Functional Plasticity of Innate Immune Cells*,” eleven contributions were brought together to highlight recent advances and perspectives on the roles of innate lymphoid cells (ILCs), innate-like T cells, neutrophils, macrophages, monocytes, and dendritic cells in regulated cell death, infectious diseases, cancer, and other pathological conditions.

Maresca et al. investigate the role of ILCs and the cytokine IL-18 as potential biomarkers in thymic epithelial tumors. They identified a significant expansion of circulating ILCs, particularly the ILC1 subset, in patients with active disease and more aggressive carcinomas. Elevated serum IL-18 levels were also found to accompany tumor growth, although a functional dissociation was observed in the production of type 1 immunity effector cytokines. These findings suggest that monitoring these cell populations and cytokines may improve patient stratification and clinical follow-up. Extending the discussion of ILC dysregulation, Jiang et al. review how natural killer (NK) cell imbalance acts as a critical link between reproductive and metabolic dysfunction in polycystic ovary syndrome (PCOS). Whereas peripheral blood NK cells are frequently increased, reflecting chronic low-grade inflammation, uterine NK cells are reduced and exhibit functional impairments that compromise endometrial receptivity and embryo implantation. Androgen excess and insulin resistance further exacerbate this condition by altering the distribution of these cells and promoting the emergence of pro-inflammatory subsets.

Two studies evaluate innate-like T cells in different conditions. Degeorges et al. analyze how melanoma disrupts the homeostasis of invariant natural killer T (iNKT) cells, essential components of immune surveillance, by altering their phenotype and metabolism. The authors found that these cells accumulate within tumors in a sex- and lesion site-dependent manner and are frequently associated with unfavorable clinical outcomes. Within the tumor microenvironment, iNKT cells exhibit low metabolic activity and reduced reliance on glycolysis, thereby impairing their antitumor efficacy. These findings provide a rationale for novel therapeutic strategies aimed at restoring the functional fitness of these cells through metabolic modulation and the blockade of exhaustion receptors. In the context of infectious disease, Song et al. investigate the relationship between γδ T cells and *Mycobacterium tuberculosis* infection. Through RNA sequencing analyses, the authors demonstrate that a heat-resistant antigen derived from *M. tuberculosis* (Mtb-HAg) induces the expression of cytotoxicity-related genes, including those encoding perforin and granzyme B, in γδ T cells, enabling them to eliminate infected macrophages and restrict bacterial replication. The BTN3A1 protein plays a key role in this process. Thus, the study elucidates crucial molecular mechanisms of innate immunity that may be harnessed to overcome the limitations of conventional drug-based therapies.

Two studies highlighted innate immune cells in the context of HIV infection and prevention. Bothwell et al. demonstrate that monocyte-derived dendritic cells exposed to type-1 inflammatory signals exhibit increased expression of Siglec-1, which functions as a critical receptor for viral capture and dissemination. This process is enhanced by interactions involving CD40L. Furthermore, the study reveals that these dendritic cells release the chemokine CCL20, thereby increasing the susceptibility of neighboring CD4^+^ T cells to infection. Shifting from cellular dysfunction to innate immune modulation during HIV prevention, Jameson et al. investigate how daily HIV pre-exposure prophylaxis (PrEP) affects the immune system of HIV-seronegative individuals. They found that tenofovir and emtricitabine induce a pro-inflammatory state by increasing monocyte activation and enhancing cytokine production following bacterial stimulation. In addition to these functional changes, PrEP promotes metabolic reprogramming in monocytes and other cell populations, reducing their reliance on glucose. Although PrEP is highly effective in preventing HIV infection, these findings suggest that it exerts secondary immunomodulatory effects that may influence chronic inflammation.

Two studies examine the relationship between distinct forms of cell death and macrophages. Jiang et al. demonstrate the role of nicotinamide phosphoribosyltransferase (NAMPT) in copper-dependent cell death, known as cuproptosis, during the progression of idiopathic pulmonary fibrosis (IPF). NAMPT-positive fibroblasts exhibit greater cuproptosis activity and maintain extensive communication with M2 macrophages through the complement C3–ITGB2 axis. This cellular interaction reinforces the profibrotic microenvironment, accelerating the severe tissue remodeling characteristic of the disease. In a review article, Xu et al. explore the role of heme oxygenase-1 (HO-1) as a regulator of ferroptosis in atherosclerosis. They describe how this mechanism specifically affects macrophages, discussing both canonical pathways and atypical processes, such as erythrophagocytosis, that contribute to cardiovascular disease progression. The role of macrophages is also discussed from an osteoimmunological perspective by Mierzejewska et al., who review the relationship between the physicochemical properties of biomaterials and the activation of macrophages and cytokine networks.

Neutrophils are described in two review articles. McGarrity-Cottrell et al. examine the fundamental roles of these cells in the immune system, emphasizing their dual functions in cancer and discussing neutrophil-based therapeutic applications across different fields. Yin et al., meanwhile, analyze how the hypoxic microenvironment acts as a regulator of immune-mediated venous thrombosis, highlighting the roles of neutrophils, monocytes/macrophages, and endothelial cells. This review establishes the hypoxia–immunity axis as a central target for novel therapeutic and preventive strategies against thrombosis.

Collectively, the studies featured in this Research Topic demonstrate that innate and innate-like immune cells are not restricted to fixed or classically defined functions but instead display remarkable context-dependent plasticity across cancer, infection, metabolic dysfunction, regulated cell death, and tissue remodeling. By identifying novel cellular states, molecular pathways, and potential biomarkers, these contributions expand our understanding of innate immunity and highlight opportunities for the development of more precise diagnostic and therapeutic strategies. Further experimental and clinical validation will be essential to translate these emerging insights into effective interventions.

